# Œsophagotomy

**Published:** 1879-08

**Authors:** 


### CASE OF ŒSOPHAGOTOMY.

Dr. Atherton, in the Boston *Medical and Surgical Journal*, reports a successful operation of œsophagotomea externa for a foreign body.

The patient was an old lady, 70 years of age, who at noon, August 28, 1877, while eating some lamb, swallowed a piece of bone, which stuck in her throat and caused a good deal of choking. She succeeded by external manipulations in working the foreign body down as far as the lower part of the neck. As all trials of either withdrawing the same or pushing it down into the stomach failed, and as the sufferings were severe, œsophagotomy was advised, and performed next day.

Operation: Chloroform was given for a few minutes, till she was partially anæsthetized, and then ether was substituted. An incision three or four inches long was made on the left side of the neck, at the inner side of the sterno-mastoid, but not quite so obliquely as that muscle runs. The anterior jugular vein lay so much in the way that it was divided, and bleeding from it controlled by torsion. After getting through the superficial fascia and platysma muscle, there came into view the anterior belly of the omo-hyoid, which with the sterno-mastoid and carotid vessels was drawn to outer side. By use of director and handle of knife the posterior part of the trachea was reached. Now a sponge probang was passed through the mouth into the œsophagus, till it brought up against the obstruction. The sponge could be felt somewhat indistinctly at the bottom of the wound, and, after considerable searching, just below it and at the lower end of the incision, was discovered a hard body, with a rather sharp outline. A slight touch of the knife brought into view the upper end of a piece of bone, which was seized by forceps and extracted. It proved to be a portion of lamb's rib, having obliquely cut ends, and measuring rather more than one and one-half inches from tip to tip. It lay nearly straight up and down in the œsophagus, but its rough, jagged ends, prevented its being readily dislodged. I omitted to mention that upon coming down upon the deep tissues I found them swollen, and the parts about the œsophagus infiltrated with what appeared to be sero-purulent matter. Also some bubbles of gas escaped before I cut into the œsophagus. The wound was sopped with carbolic acid and water, one to eight. Also three stitches were put in the upper part of the incision. The rest was left open. Carbolized oil dressing. The first three days the patient was nourished with enemata, but after that time by injection of milk, broth, egg-nog and brandy through a tube, introduced into the stomach. Some sloughing of the wound occurred, but, on the whole, the case progressed favorably, and the introduction of the tube was omitted on the 22d of September. October 2d wound entirely healed. The author would in another case not allow nourishment to be taken as soon by the mouth, believing that the wound was made unhealthy by its contact as it escaped externally.

